# Supplementary material for: Prevalence of metabolic syndrome among Iranian postmenopausal females: A systematic review and meta-analysis
Source: PLoS One. 2025 Dec 16;20(12):e0338599. doi: 10.1371/journal.pone.0338599 (PMC12707683; doi:10.1371/journal.pone.0338599)
Supplement: S1 File — (DOCX) [file pone.0338599.s001.docx]

**Search Syntax**

- Lines colored in red indicate terms retrieved by free-text methods.
- A two-term search strategy was employed, combining one term related to metabolic syndrome and one term associated with menopause, using simplified search interfaces (such as Civilica, Scholar, MagIran, etc.) in both English and Persian.

***PUBMED***

(“Metabolic Syndrom*” OR (Syndrom* AND Metabolic) OR “Reaven Syndrome X” OR (“Syndrome X” AND Reaven) OR “Metabolic Syndrome X” OR “Insulin Resistance Syndrome X” OR “Metabolic Cardiovascular Syndrome” OR (“Cardiovascular Syndrom*” AND Metabolic) OR (Syndrom* AND “Metabolic Cardiovascular”) OR (“Syndrome X” AND “Insulin Resistance”) OR “Metabolic X Syndrome” OR (Syndrom* AND “Metabolic X”) OR (“X Syndrom*” AND Metabolic) OR (“Syndrome X” AND Metabolic) OR “Dysmetabolic Syndrome X” OR (“Syndrome X” AND Dysmetabolic) OR “Cardiometabolic Syndrom*” OR (Syndrom* AND Cardiometabolic) OR “cardiometabolic profile” OR “MetS” OR “CMS” OR “Metabolic triad syndrom*” OR “Metabolic dysfunction syndrom*” OR “Metabolic disorder syndrome” OR “deadly quartet” OR “cardiometabolic impairment”) **AND** (“menopaus*” OR “postmenopaus*” OR “Permanent cessation of mens*” OR “last menstrual period” OR (“Change of Life” AND (Female OR woman OR women)) OR “Post-menopausal Period” OR (Period AND Post-menopausal) OR “Post menopausal Period” OR “Post-Menopaus*” OR “Post Menopaus*” OR “Female midlife transition” OR “After menopaus*”) **AND** (Iran[tiab] OR Iran[ad] OR Iran[pl])

***EMBASE***

('Metabolic Syndrom*' OR (Syndrom* AND Metabolic) OR 'Reaven Syndrome X' OR ('Syndrome X' AND Reaven) OR 'Metabolic Syndrome X' OR 'Insulin Resistance Syndrome X' OR 'Metabolic Cardiovascular Syndrome' OR ('Cardiovascular Syndrom*' AND Metabolic) OR (Syndrom* AND 'Metabolic Cardiovascular') OR ('Syndrome X' AND 'Insulin Resistance') OR 'Metabolic X Syndrome' OR (Syndrom* AND 'Metabolic X') OR ('X Syndrom*' AND Metabolic) OR ('Syndrome X' AND Metabolic) OR 'Dysmetabolic Syndrome X' OR ('Syndrome X' AND Dysmetabolic) OR 'Cardiometabolic Syndrom*' OR (Syndrom* AND Cardiometabolic) OR 'cardiometabolic profile' OR 'MetS' OR 'CMS' OR 'Metabolic triad syndrome*' OR 'Metabolic dysfunction syndrom*' OR 'Metabolic disorder syndrome' OR 'deadly quartet' OR 'cardiometabolic impairment') **AND** ('menopaus*' OR 'postmenopaus*' OR 'Permanent cessation of mens*' OR 'last menstrual period' OR ('Change of Life' AND (Female OR woman OR women)) OR 'Post-menopausal Period' OR (Period AND Post-menopausal) OR 'Post menopausal Period' OR 'Post-Menopaus*' OR 'Post Menopaus*' OR 'Female midlife transition' OR 'After menopaus*') **AND** (Iran:ti,ab,kw OR Iran:ff OR Iran:cy)

***SCOPUS***

(TITLE-ABS-KEY(“Metabolic Syndrom*”) OR (TITLE-ABS-KEY (Syndrom*) AND TITLE-ABS-KEY(Metabolic)) OR TITLE-ABS-KEY(“Reaven Syndrome X”) OR (TITLE-ABS-KEY(“Syndrome X”) AND TITLE-ABS-KEY(Reaven)) OR TITLE-ABS-KEY(“Metabolic Syndrome X”) OR TITLE-ABS-KEY(“Insulin Resistance Syndrome X”) OR TITLE-ABS-KEY(“Metabolic Cardiovascular Syndrome”) OR (TITLE-ABS-KEY(“Cardiovascular Syndrom*”) AND TITLE-ABS-KEY(Metabolic)) OR (TITLE-ABS-KEY(Syndrom*) AND TITLE-ABS-KEY(“Metabolic Cardiovascular”)) OR (TITLE-ABS-KEY(“Syndrome X”) AND TITLE-ABS-KEY(“Insulin Resistance”)) OR TITLE-ABS-KEY(“Metabolic X Syndrome”) OR (TITLE-ABS-KEY(Syndrom*) AND TITLE-ABS-KEY(“Metabolic X”)) OR (TITLE-ABS-KEY(“X Syndrom*”) AND TITLE-ABS-KEY(Metabolic)) OR (TITLE-ABS-KEY(“Syndrome X”) AND TITLE-ABS-KEY(Metabolic)) OR TITLE-ABS-KEY(“Dysmetabolic Syndrome X”) OR (TITLE-ABS-KEY(“Syndrome X”) AND TITLE-ABS-KEY(Dysmetabolic)) OR TITLE-ABS-KEY(“Cardiometabolic Syndrom*”) OR (TITLE-ABS-KEY(Syndrom*) AND TITLE-ABS-KEY(Cardiometabolic)) OR TITLE-ABS-KEY(“cardiometabolic profile”) OR TITLE-ABS-KEY(“MetS”) OR TITLE-ABS-KEY(“CMS”) OR TITLE-ABS-KEY(“Metabolic triad syndrom*”) OR TITLE-ABS-KEY(“Metabolic dysfunction syndrom*”) OR TITLE-ABS-KEY(“Metabolic disorder syndrome”) OR TITLE-ABS-KEY(“deadly quartet”) OR TITLE-ABS-KEY(“cardiometabolic impairment”)) **AND** (“menopaus*” OR “postmenopaus*” OR “Permanent cessation of mens*” OR “last menstrual period” OR (“Change of Life” AND (Female OR woman OR women)) OR “Post-menopausal Period” OR (Period AND Post-menopausal) OR “Post menopausal Period” OR “Post-Menopaus*” OR “Post Menopaus*” OR “Female midlife transition” OR “After menopaus*”) **AND** (TITLE-ABS-KEY(Iran) OR AFFIL(Iran))

***Web of Science***

ALL=((Metabolic Syndrom*) OR (Syndrom* AND Metabolic) OR (Reaven Syndrome X) OR ((Syndrome X) AND Reaven) OR (Metabolic Syndrome X) OR (Insulin Resistance Syndrome X) OR (Metabolic Cardiovascular Syndrome) OR ((Cardiovascular Syndrom*) AND Metabolic) OR (Syndrom* AND (Metabolic Cardiovascular)) OR ((Syndrome X) AND (Insulin Resistance)) OR (Metabolic X Syndrome) OR (Syndrom* AND (Metabolic X)) OR ((X Syndrom*) AND Metabolic) OR ((Syndrome X) AND Metabolic) OR (Dysmetabolic Syndrome X) OR ((Syndrome X) AND Dysmetabolic) OR (Cardiometabolic Syndrom*) OR (Syndrom* AND Cardiometabolic) OR (cardiometabolic profile) OR (MetS) OR (CMS) OR (Metabolic triad syndrom*) OR (Metabolic dysfunction syndrom*) OR (Metabolic disorder syndrome) OR (deadly quartet) OR (cardiometabolic impairment))

**AND**

ALL=(menopaus* OR postmenopaus* OR (Permanent cessation of mens*) OR (last menstrual period) OR ((Change of Life) AND (Female OR woman OR women)) OR (Post-menopausal Period) OR (Period AND Post-menopausal) OR (Post menopausal Period) OR (Post-Menopaus*) OR (Post Menopaus*) OR (Female midlife transition) OR (After menopaus*))

**AND**

ALL=(Iran)
