# Supplementary material for: Prevalence of metabolic syndrome among Iranian postmenopausal females: A systematic review and meta-analysis
Source: PLoS One. 2025 Dec 16;20(12):e0338599. doi: 10.1371/journal.pone.0338599 (PMC12707683; doi:10.1371/journal.pone.0338599)
Supplement: S2 File — (DOCX) [file pone.0338599.s002.docx]

On 22 June 2014, Iran's thirty-one provinces were reclassified into the following five regional divisions following a decree by the Iranian Ministry of Interior. This restructuring was based on factors such as provincial adjacency, geographical proximity, and shared regional characteristics.

- Region 1:
- Alborz province
- Golestan province
- Mazandaran province
- Qazvin province
- Qom province
- Semnan province
- Tehran province
- Region 2:
- Bushehr province
- Chaharmahal and Bakhtiari province
- Fars province
- Hormozgan province
- Isfahan province
- Kohgiluyeh and Boyer-Ahmad province
- Region 3:
- Ardabil province
- East Azerbaijan province
- Gilan province
- Kurdistan province
- West Azerbaijan province
- Zanjan province
- Region 4:
- Hamadan province
- Ilam province
- Kermanshah province
- Khuzestan province
- Lorestan province
- Markazi province
- Region 5:
- Kerman province
- North Khorasan province
- Razavi Khorasan province
- Sistan and Baluchestan province
- South Khorasan province
- Yazd province
