# Supplementary material for: Prevalence of metabolic syndrome among Iranian postmenopausal females: A systematic review and meta-analysis
Source: PLoS One. 2025 Dec 16;20(12):e0338599. doi: 10.1371/journal.pone.0338599 (PMC12707683; doi:10.1371/journal.pone.0338599)
Supplement: S3 File — (DOCX) [file pone.0338599.s003.docx]

**Table S1.** Results of the quality assessment with JBI tool.

| **Study** | **JBI Critical Appraisal Checklist for Studies Reporting Prevalence Data** | | | | | | | | | **Score** | **Overall** |
| --- | --- | --- | --- | --- | --- | --- | --- | --- | --- | --- | --- |
|  | Q1 | Q2 | Q3 | Q4 | Q5 | Q6 | Q7 | Q8 | Q9 |  |  |
| Abbasi 2017 | No | No | Unclear | Yes | Yes | Yes | Yes | Yes | Unclear | 5 | Moderate |
| Ayni 2007 | Yes | Yes | Yes | Yes | Yes | Yes | Yes | Yes | Unclear | 8 | High |
| Heidari 2015 | Yes | Yes | Yes | Yes | Yes | Yes | Yes | Yes | Unclear | 8 | High |
| Nabipour 2010 | Yes | Yes | Yes | Yes | Yes | Yes | Yes | Yes | Unclear | 8 | High |
| Delavar 2009 | Yes | Yes | Yes | Yes | Yes | Yes | Yes | Yes | Unclear | 8 | High |
| Ebrahimpour 2010 | Yes | Yes | Yes | Yes | Yes | Yes | Yes | Yes | Unclear | 8 | High |
| Eshtiaghi 2010 | Yes | Unclear | Yes | Yes | Yes | Yes | Yes | Yes | Unclear | 7 | High |
| Farahmand 2017 | Yes | Yes | Yes | Yes | Yes | Yes | Yes | Yes | Yes | 9 | High |
| Soleimani 2018 | Yes | Yes | Yes | Yes | Yes | Yes | Yes | Yes | Yes | 9 | High |
| Namazi Shabestari 2016 | No | No | Unclear | Yes | Yes | Yes | Yes | Yes | Unclear | 5 | Moderate |
| Maharlouei 2013 | No | Unclear | Yes | Yes | Yes | Yes | Yes | Yes | Unclear | 6 | Moderate |
| Marjani 2012 | Unclear | Unclear | Unclear | Yes | Yes | Yes | Yes | Yes | Unclear | 5 | Moderate |
| Montazeri 2023 | Yes | Yes | Yes | Yes | Yes | Yes | Yes | Yes | Unclear | 8 | High |
| Moradi 2024 | Yes | Unclear | Yes | Yes | Yes | Yes | Yes | Yes | Unclear | 7 | High |
| Naghipour 2022 | Yes | Yes | Yes | Yes | Yes | Yes | Yes | Yes | Unclear | 8 | High |
| Nakhjavani 2014 | No | No | Yes | Yes | Yes | Yes | Yes | Yes | Unclear | 6 | Moderate |
| Sayahi 2015 | Yes | Yes | Unclear | Yes | Yes | Yes | Yes | Yes | Unclear | 7 | High |
| Shahvazi 2016 | No | Yes | Yes | Yes | Yes | Yes | Yes | Yes | Unclear | 7 | High |
| Zareei 2022 | Yes | Yes | Yes | Yes | Yes | Yes | Yes | Yes | Unclear | 8 | High |
| Bakhtiari 2018 | No | No | Yes | Yes | Yes | Yes | Yes | Yes | Unclear | 6 | Moderate |
| Rabiei 2021 | Yes | Yes | Yes | Yes | Yes | Yes | Yes | Yes | Unclear | 8 | High |
| Sadat 2015 | No | Yes | Unclear | Yes | Yes | Yes | Yes | Yes | Unclear | 6 | Moderate |
| Saeedi 2023 | Yes | Yes | Yes | Yes | Yes | Yes | Yes | Yes | Unclear | 8 | High |
| Ziaei 2011 | Unclear | No | Unclear | Yes | Yes | Yes | Yes | Yes | Unclear | 5 | Moderate |

**Q1.** Was the sample frame appropriate to address the target population? / **Q2.** Were study participants sampled in an appropriate way? / **Q3.** Was the sample size adequate? / **Q4.** Were the study subjects and the setting described in detail? / **Q5.** Was the data analysis conducted with sufficient coverage of the identified sample? / **Q6.** Were valid methods used for the identification of the condition? / **Q7.** Was the condition measured in a standard, reliable way for all participants? / **Q8.** Was there appropriate statistical analysis? / **Q9.** Was the response rate adequate, and if not, was the low response rate managed appropriately?
