# Supplementary material for: Prevalence of metabolic syndrome among Iranian postmenopausal females: A systematic review and meta-analysis
Source: PLoS One. 2025 Dec 16;20(12):e0338599. doi: 10.1371/journal.pone.0338599 (PMC12707683; doi:10.1371/journal.pone.0338599)
Supplement: S4 File — (DOCX) [file pone.0338599.s004.docx]

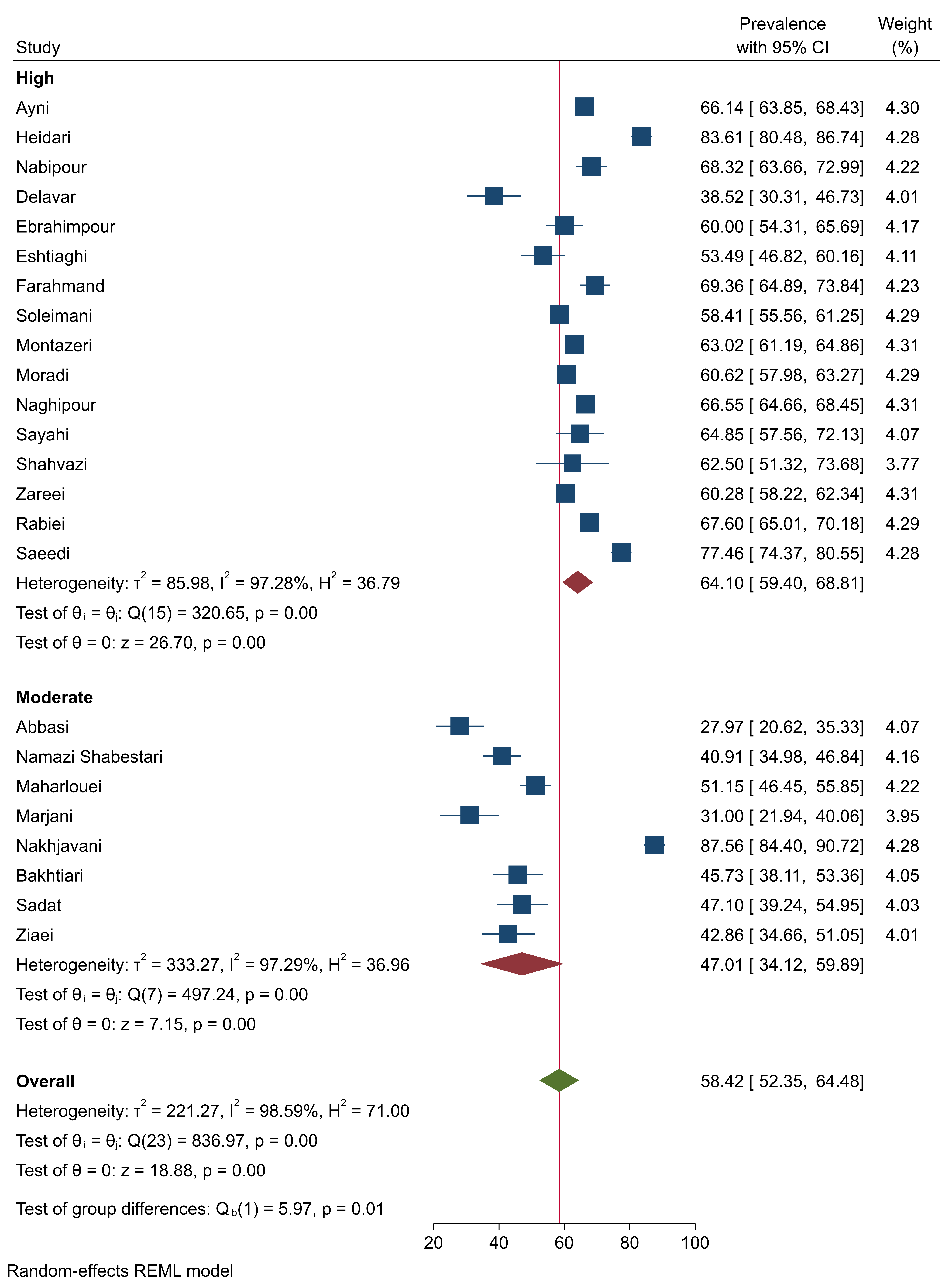


**Figure S1.** Forest plot demonstrating prevalence of MetS among Iranian postmenopausal females in both individual primary studies and the overall estimate with 95%CI categorized by study quality.


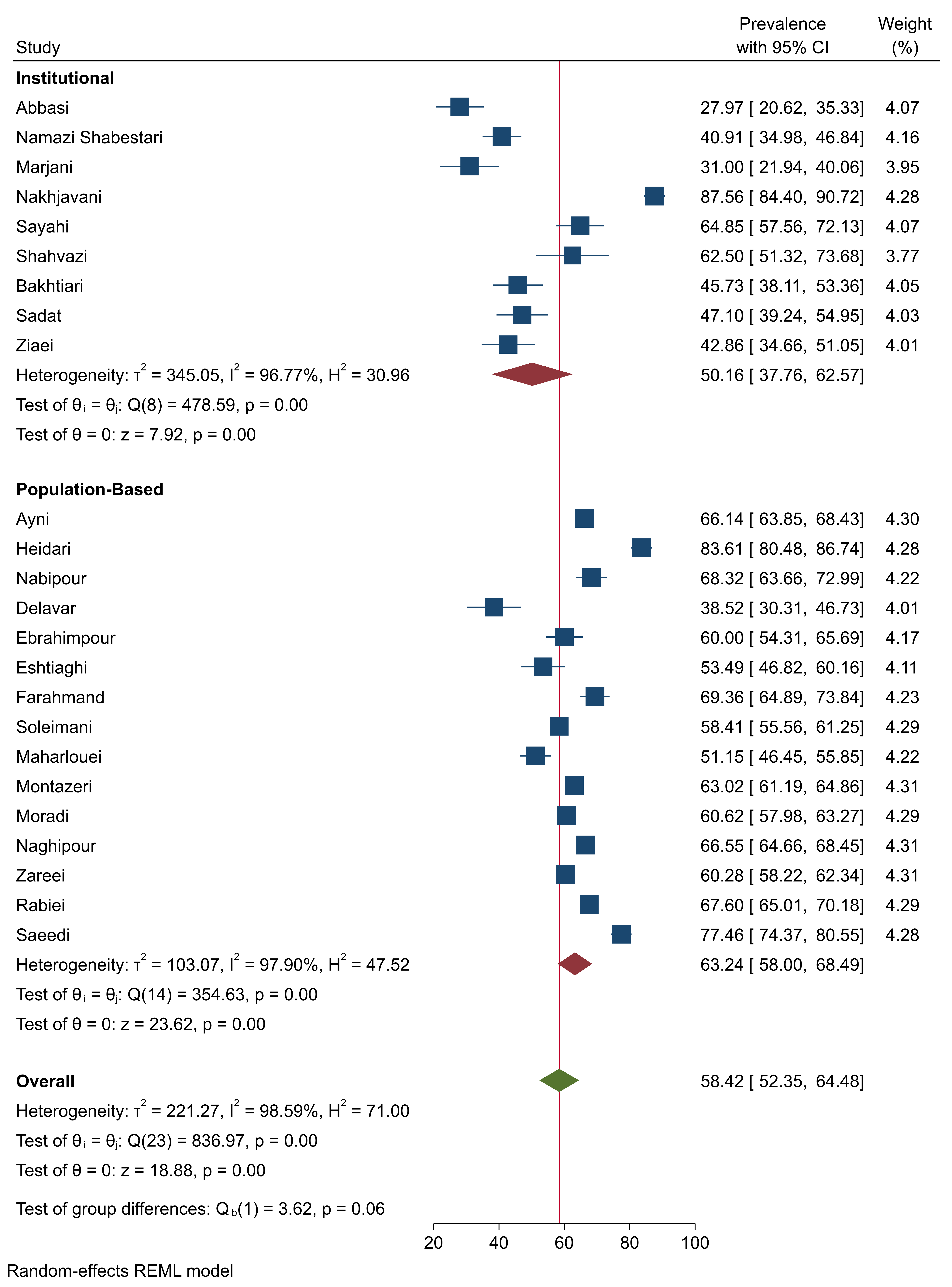


**Figure S2.** Forest plot demonstrating prevalence of MetS among Iranian postmenopausal females in both individual primary studies and the overall estimate with 95%CI categorized by study setting.


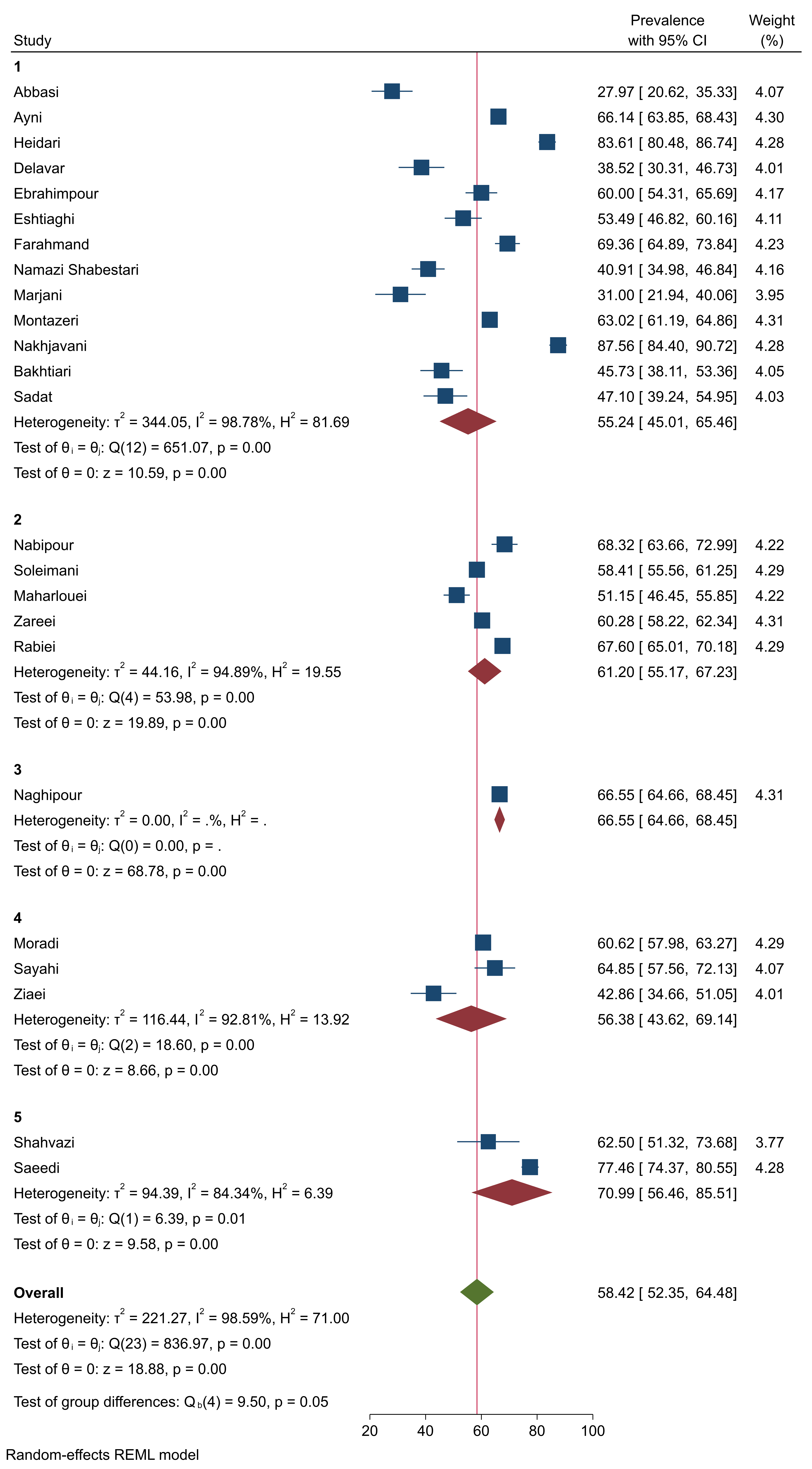


**Figure S3.** Forest plot demonstrating prevalence of MetS among Iranian postmenopausal females in both individual primary studies and the overall estimate with 95%CI categorized by study region (based on Iranian Ministry of Interior classification).


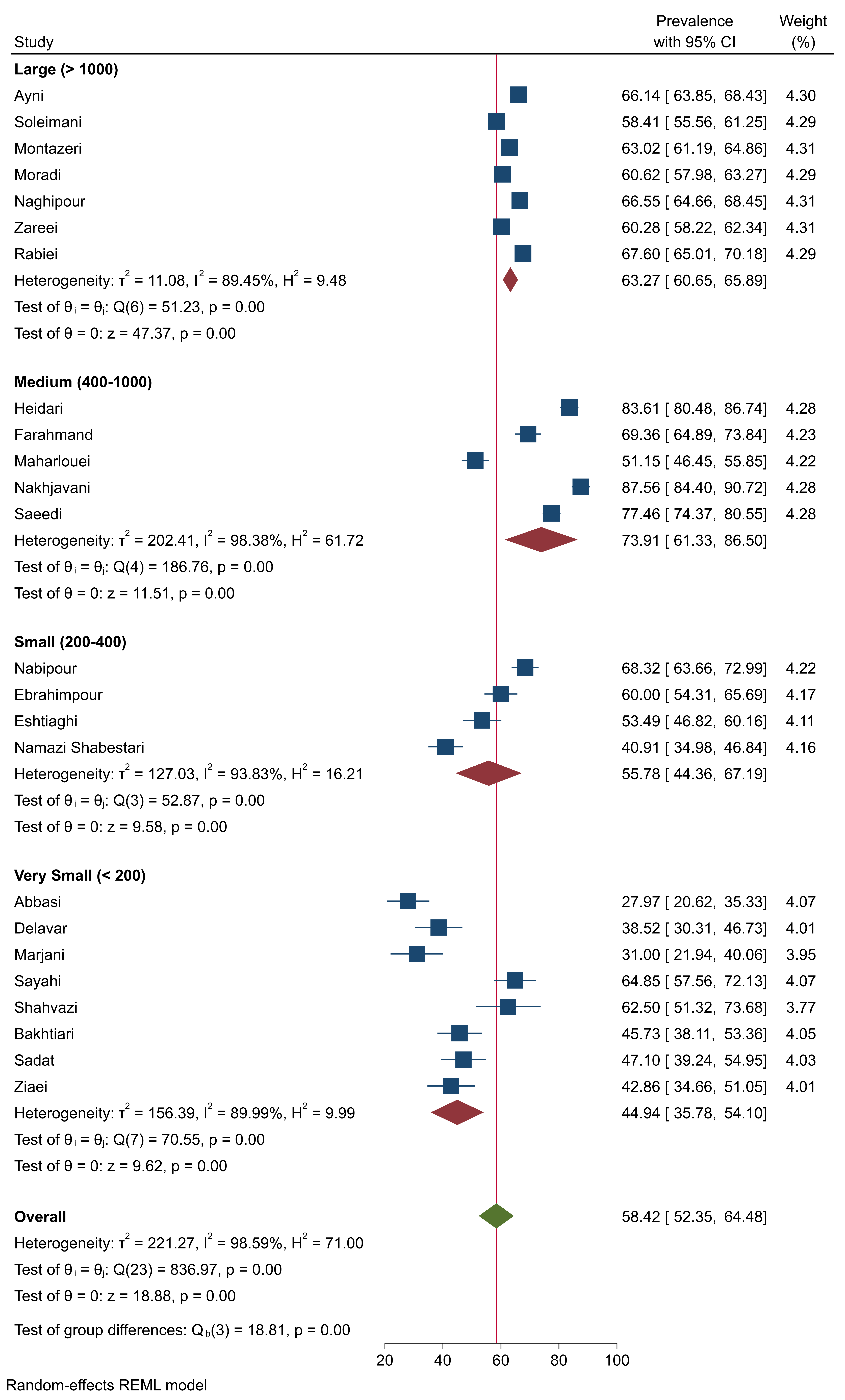


**Figure S4.** Forest plot demonstrating prevalence of MetS among Iranian postmenopausal females in both individual primary studies and the overall estimate with 95%CI categorized by study sample size frame.


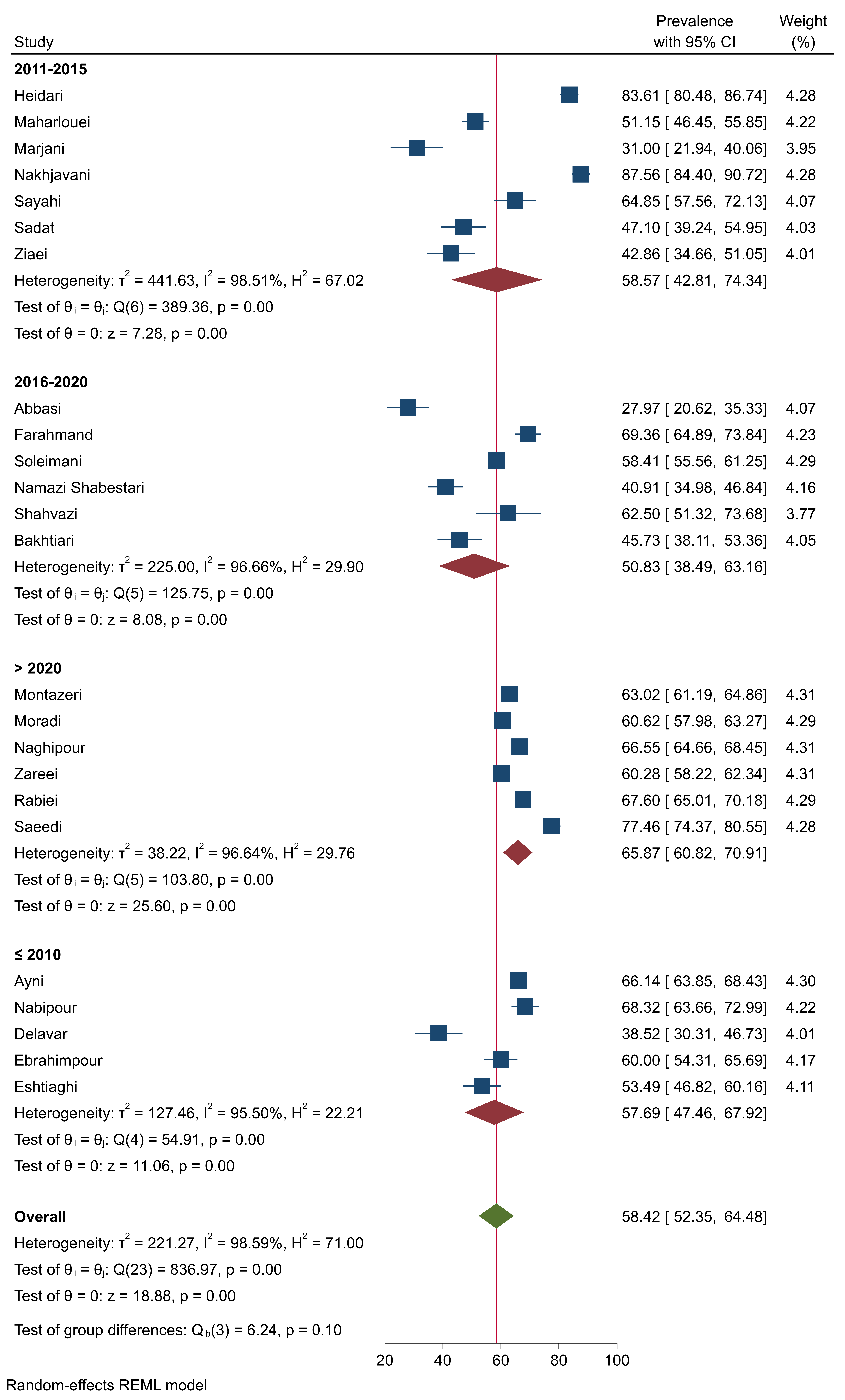


**Figure S5.** Forest plot demonstrating prevalence of MetS among Iranian postmenopausal females in both individual primary studies and the overall estimate with 95%CI categorized by study year.


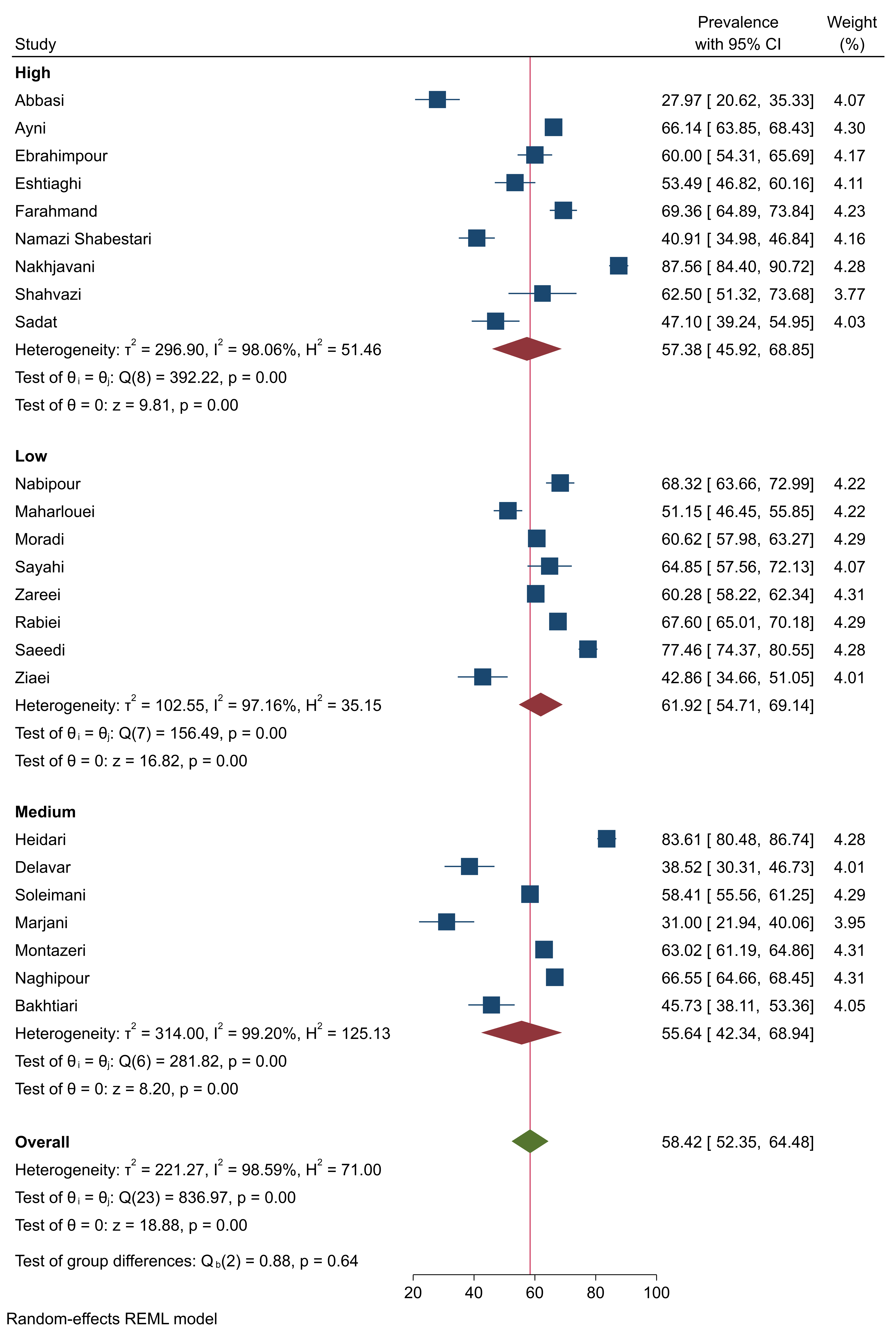


**Figure S6.** Forest plot demonstrating prevalence of MetS among Iranian postmenopausal females in both individual primary studies and the overall estimate with 95%CI categorized by healthcare price index category.


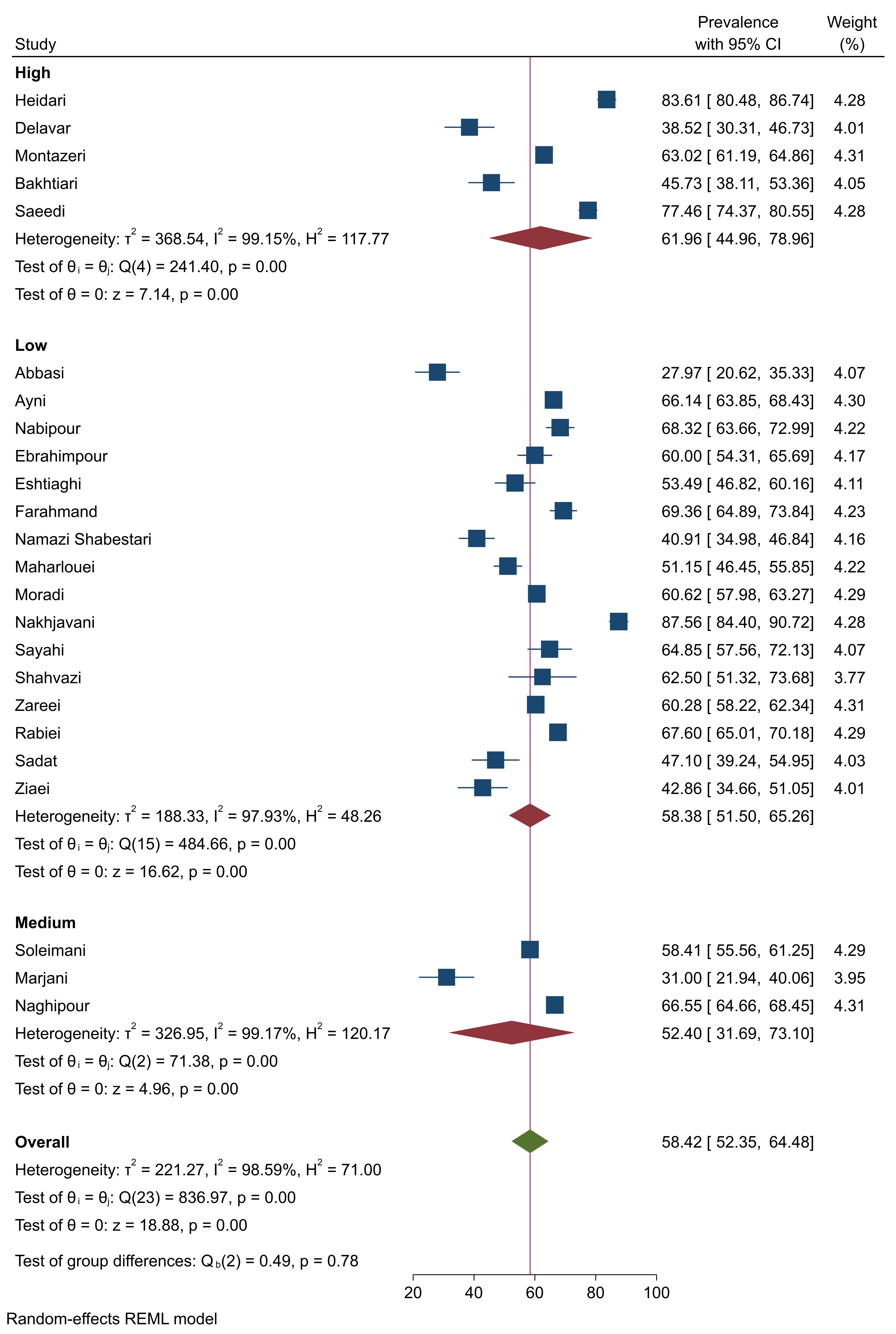


**Figure S7.** Forest plot demonstrating prevalence of MetS among Iranian postmenopausal females in both individual primary studies and the overall estimate with 95%CI categorized by primary care center density category.
