# Supplementary material for: Prevalence of metabolic syndrome among Iranian postmenopausal females: A systematic review and meta-analysis
Source: PLoS One. 2025 Dec 16;20(12):e0338599. doi: 10.1371/journal.pone.0338599 (PMC12707683; doi:10.1371/journal.pone.0338599)
Supplement: Attachment — Submitted filename: Response to Reviewers R1.docx [file pone.0338599.s006.docx]

Dear ***PLoS One*** Editorial Team,

We would like to express our sincere gratitude to you and your team of reviewers for the time and consideration you gave to our manuscript titled “**Prevalence of Metabolic Syndrome among Iranian Postmenopausal Females: a Systematic Review and Meta-Analysis**”. Your expertise and attention to detail were invaluable in helping us to refine our research and present it in a way that is clear and meaningful. We are especially grateful for the helpful comments and feedbacks provided by the reviewer. Reviewer’s insightful critiques and suggestions allowed us to improve the quality of our research and strengthen the overall presentation of our work. We appreciate the opportunity you have given us to share our research with the academic community. Below are the answers to your comments. Furthermore, changes in the manuscript have been highlighted in yellow. We hope you find it helpful.

**Editor**
**Comment:** Please revise Flowchart in accordance with PRISMA 2020 guideline.

Answer: Thank you for your feedback. We have revised this flowchart.

**Reviewer #1**

**Comment:** This remarkable and comprehensive work reports on the far-reaching consequences of metabolic syndrome in women after they enter the monophase, because it represents the main burden in the field of NCDs on the different dimensions of health. The results can highlight the importance of this risk factor and its potential impact on women after the fertility period in Iran. I have made a few comments on this manuscript, consideration of which may improve the quality and clarity of your reports:

In the abstract, you should specify the exact time frame for the literature and database search, e.g. (August 2010 to April 2025).

Answer: Thank you for your comment. Databases were searched from their dates of inception until April 2025. This has been specified in the revised abstract.

**Comment:** In the introduction, in lines 54 and 55, you mentioned the Burdens of MetS in Iran, for more clarity you need to give the year of this rate and statistics.

Answer: Thank you for your feedback. This statistic was the result of an updated meta-analysis by Moradkhani et al. which was published on 2025. This has been indicated on line 58.

**Comment:** Two more sentences about the components and criteria for MetS in the first paragraph of the discussion could be informative for readers and researchers.

Answer: The authors gratefully acknowledge the reviewer's suggestion. Although it is standard practice for the opening paragraph of the Discussion to summarize the principal findings, we agree that providing 2-3 sentences on the components and diagnostic criteria for MetS would enhance the manuscript's clarity for readers. A paragraph to this effect has therefore been added as the second paragraph of the Discussion section.

**Comment:** The exclusion criteria (number 4) for study selection should be rewritten and simplified, e.g. what you mean by “overlapping population”

Answer: We thank the reviewer for this valuable suggestion. To improve clarity and precision, we have rewritten the fourth exclusion criterion in the manuscript. This criterion explicitly prevents the inclusion of multiple reports from the same parent study or cohort, thereby avoiding the double-counting of participants and the associated statistical bias of multiplicity in the meta-analysis.

**Comment:** In the data extraction section (line;146), “number of postmenopausal females” should be replaced by “proportion of females….”.

Answer: Thank you for your feedback. This has been rewritten.

**Comment:** In the section on participants, you should specify the minimum age of female participants or the age of participants, as the start of the monophase in different population groups.

Answer: We thank the reviewer for this insightful comment. The reviewer rightly points out that the natural age at menopause onset can vary across populations. In our study, the operational definition for a "postmenopausal female" was explicitly based on the clinical criterion of "cessation of menstrual period for at least 12 months" (as stated in the *Participants* section), rather than on a specific age threshold. This definition accurately identifies the postmenopausal state regardless of the population's average age at menopause. However, to fully address the reviewer's concern and provide greater methodological clarity, we have now added the following sentence to the *Participants* subsection in the revised manuscript:

*"This definition based on amenorrhea, rather than a predetermined age cutoff, was employed to accurately capture the postmenopausal status across all included studies, accounting for potential variations in the natural age at menopause within the Iranian population."*

Furthermore, we have extracted and presented the mean chronological age and menopausal age of participants from each included study (if reported) in Table 1, which provides readers with a picture of the age profile of the synthesized population.

**Comment:** Regarding the significant contribution of participant age to heterogeneity, a subgroup analysis based on age has been suggested to improve the significance of the pooled prevalence you report.

Answer: We thank the reviewer for this valuable suggestion. We agree that a subgroup analysis based on age would be highly informative. As the reviewer noted, our meta-regression confirmed that participant age was a significant source of heterogeneity (P: 0.038). However, performing a reliable subgroup analysis was precluded by a critical data limitation in the primary studies. Specifically, only 16 of the 24 included studies reported the mean age of their postmenopausal participants. Among these, the mean age ranged from 53.98 to 69.14 years. Categorizing these studies into discrete age groups (e.g., <55 vs. ≥55 years) would be methodologically unsound, as the minimum and maximum ages and the underlying age distributions across these studies are unknown and likely overlap significantly.

To address this important limitation transparently, we have now added the following statement to the "Limitations and Future Directions" section of the revised manuscript:

*"Furthermore, while meta-regression identified participant age as a significant source of heterogeneity, a subgroup analysis by age was not feasible due to inconsistent and incomplete reporting of age data across primary studies. Future primary studies should report age-stratified prevalence data to enable more granular meta-analyses."*

We acknowledge that an Individual Participant Data (IPD) meta-analysis would be the ideal approach to overcome this limitation, though it is often not feasible. We believe that explicitly stating this limitation in the manuscript, as now done, appropriately highlights the issue and guides future research, as the reviewer intended.

**Comment:** The periods of the studies should also be considered in your subgroup analysis. Because if there is a trend in prevalence over time, it should be discussed further.

Answer: Thank you for your suggestion. We have added subgroup analysis results based on study year (categorized as studies on and before 2010, 2011 to 2015, 2016 to 2020, and after 2020) which did not yield specific trends in prevalence over time.

**Reviewer #2**

Dear authors,

The manuscript highlight the need for high quality evidence regarding the prevalence of metabolic syndrome in postmenopausal women in Iran - a subset of the general population which is often neglected despite an optimal availability for delivery primary primary health care services. The following recommendations for revisions are made in hopes that the final manuscript may better address the study aims:

**Comment 1.** A significant amount of time has lapsed since the previously mentioned systematic reviews of prevalence, and results from the PERSIAN cohort - although of high epidemiological value - are not the sole reason for the need of an updated appraisal of the evidence. Please highlight other newly published evidence in the introduction.

Answer: We thank the reviewer for this insightful comment. We completely agree that the need for an updated synthesis extends beyond the inclusion of the PERSIAN cohort. To address this point thoroughly, we have revised the Introduction to more comprehensively outline the gaps left by previous reviews and the breadth of new evidence now available.

Since the last major reviews in 2018, a substantial number of new studies have been published. We now mention that our search identified several recent, high-quality population-based cohorts (such as the Tabari, Hoveyzeh, Guilan, and Fasa Cohort Studies, among others) which were not available for prior analyses. Also, our review employs more rigorous and contemporary methodological standards (e.g., comprehensive search, subgroup analysis, meta-regression) to address the heterogeneity that limited earlier efforts. Below, we present to you several advantages of our paper compared to prior reviews in this field, and outline the most crucial limitations of each previous review and demonstrate how our study enhances methodological rigor and contributes to advancing the field. These were previously mentioned in the Cover Letter during the submission:

- **Review No 1.** Ebtekar F, Dalvand S, Gheshlagh RG. The prevalence of metabolic syndrome in postmenopausal women: A systematic review and meta-analysis in Iran. Diabetes & Metabolic Syndrome: Clinical Research & Reviews. 2018 Nov 1;12(6):955-60.
- *Issue 1: Suboptimal Search Strategy and Syntax*

Search strategy and syntax are not optimal. We found several key terms which were missing in the search syntax such as cardiometabolic syndrome, etc.

- *Issue 2:  Inconsistency in Diagnostic Criteria and Subgroup Analysis*

Although the previous study acknowledges variability in diagnostic criteria across primary studies and attempts to address this through subgroup analysis, such an approach inadvertently introduces methodological heterogeneity. Upon re-evaluating the included studies, we observed that many of them reported data based on the NCEP ATP III criteria, even when multiple definitions (e.g., IDF, JIS) were cited. By consistently extracting metabolic syndrome status using the NCEP ATP III criteria, our analysis minimizes methodological heterogeneity, thereby enhancing the reliability and comparability of the findings.

- *Issue 3: Missing Key Relevant Studies*

A critical examination of the previous meta-analysis reveals the exclusion of several seminal studies in this field, including Delavar (2009), Shahvazi (2016), Sadat (2015), and etc. These omissions represent a significant limitation, as these studies contain pertinent epidemiological data on metabolic syndrome prevalence in the target population. The absence of these key publications may have substantially influenced the pooled estimates and overall conclusions of the previous analysis.

- *Issue 4: Failure to Address Population Overlap in Included Studies*

A significant methodological concern in the previous meta-analysis is the inclusion of studies with overlapping populations without appropriate adjustment. Notably, the analysis incorporated multiple publications from the Tehran Lipid and Glucose Study at enrollment phase (e.g., Ziaei 2011 and Ziaei 2013; Farahmand 2013, Farahmand 2014, and Ayni 2007; Jouyandeh 2013 and Namazi Shabestari 2016) that essentially represent duplicate population samples. This oversight violates the fundamental assumption of independence required for valid meta-analytic pooling and may have artificially inflated the sample size while introducing bias in the effect estimates.

- *Issue 5: Failure to Account for Multiplicity in Data Synthesis*

The previous meta-analysis committed a critical methodological error by incorporating duplicate data from the same study population multiple times in their synthesis. A clear example of this multiplicity occurs with the Maharlouei et al. (2013) study, where prevalence estimates derived from different diagnostic criteria (NCEP ATP III and IDF) for the identical population were treated as independent datasets. This approach artificially inflates the sample size and violates the fundamental statistical principle of data independence, potentially distorting the pooled estimates and their precision.

- *Issue 6: Lack of Transparency in Quality Assessment Methodology*

The previous meta-analysis suffers from insufficient methodological transparency in its quality appraisal process. While the authors classified studies as high, medium, or low quality, they failed to provide supplementary materials documenting the rationale for individual study ratings. This opaque approach prevents critical evaluation of the quality assessment's validity and reproducibility.

- *Issue 7: Inconsistent Menopause Definition*

The prior meta-analysis failed to address significant heterogeneity in the operational definition of postmenopausal status across included studies. While some investigations defined menopause as ≥12 months of amenorrhea (consistent with WHO criteria), others employed more stringent thresholds (e.g., ≥3 years). Notably, our analysis revealed that studies reporting extended definitions also provided disaggregated data for the standard 12-month threshold.

*Overall advantages and strengths of our manuscript compared to Ebtekar et al:*

In contrast to the study by Ebtekar et al., our research employed a more robust search strategy, incorporating key terms derived from both MeSH and free-text methods. Furthermore, we expanded our search to include over 10 local and international databases and search engines, enabling the identification of several studies overlooked in their work. We also observed that nearly all included studies assessed metabolic syndrome status using either the NCEP ATP III criteria exclusively or in combination with other diagnostic criteria. To minimize methodological heterogeneity, a factor not addressed by Ebtekar et al., we exclusively extracted data based on the NCEP ATP III definition. Additionally, we meticulously evaluated eligible studies to prevent multiplicity and population overlap. To ensure methodological rigor, we performed a quality assessment using the internationally recognized Joanna Briggs Institute critical appraisal checklist for prevalence studies and provided full results in a supplementary file to enhance transparency. Moreover, we addressed methodological heterogeneity in the definition of menopause by consolidating varying criteria, resulting in a broadly defined postmenopausal population characterized by the cessation of menstruation for at least 12 consecutive months.

- **Review No 2:** Tabatabaei-Malazy O, Djalalinia S, Asayesh H, Shakori Y, Esmaeili Abdar M, Mansourian M, Mahdavi Gorabi A, Noroozi M, Qorbani M. Menopause and metabolic syndrome in the Middle East countries; a systematic review and meta-analysis study. Journal of diabetes & metabolic disorders. 2018 Dec 31;17:357-64.
- *Issue 1: Suboptimal Search Strategy and Syntax*

Search strategy and syntax are not optimal. Also, search was conducted on only three databases. We found several key terms which were missing in the search syntax such as cardiometabolic syndrome, etc.

- *Issue 2: Absence of Sensitivity Analysis*
- *Issue 3: Lack of Publication Bias Assessment*
- *Issue 4: Insufficient Exploration of Heterogeneity*

*Overall advantages and strengths of our manuscript compared to Tabatabaei-Malazy et al:*

In contrast to the study by Tabatabaei-Malazy et al., our research employed a more robust search strategy, incorporating key terms derived from both MeSH and free-text methods. Furthermore, we expanded our search to include over 10 local and international databases and search engines, enabling the identification of several studies overlooked in their work. Moreover, we: 1. conducted sensitivity analysis by leave-one-out method to assess individual study influence, 2. implemented a comprehensive publication bias assessment protocol including visual inspection of Doi plots (and the LFK index), Egger and Begg’s tests, and trim & fill method, 3. advanced the results through subgroup analysis and meta-regression.

We believe these revisions and answers to your comment provide a much stronger and more complete justification for our study, accurately reflecting the broader evolution of the evidence base as the reviewer suggested. Thank you again for your comment.

**Comment 2.** Please provide further justifications for targeting the prevalence of MetS in postmenopausal women in the manuscript introduction. Particularly, evidence regarding coverage of related services, control, and under-diagnosis - if applicable. Furthermore the introduction would benefit from inclusion of specific policies the current update might impact would impact.

Answer: We sincerely thank the reviewer for this crucial suggestion. We have thoroughly revised the Introduction to address these points. We have added a new paragraph explicitly outlining the rationale for focusing on postmenopausal women, moving beyond the biological rationale to include health systems, public health perspective, and policy perspectives. We now highlight the issue of under-diagnosis and fragmented care. We have also integrated an introduction of the specific national policies and programs that our updated prevalence data can directly inform.

**Comment 3.** Retrospective assurance of the inclusion of all studies included in previous review on this topic and the evaluation of each study's citing and cited literature is recommended for a comprehensive review - if not already performed.

Answer: We thank the reviewer for this important methodological suggestion. To address this, we did indeed perform a retrospective check of the studies included in the previous key systematic reviews by Ebtekar et al. (2018) and Tabatabaei-Malazy et al. (2018). Furthermore, as part of our search strategy, we conducted a manual review of the reference lists of all included articles and related review papers (checking "cited literature") to identify any potentially missed primary studies. However, as the reviewer astutely implies, a critical aspect of a comprehensive and *methodologically sound* review is not just the identification of studies, but their correct eligibility assessment. We found that the previous reviews contained a significant methodological flaw: *the inclusion of multiple studies that analyzed the same or heavily overlapping participant populations* (e.g., different publications from the same cohort like the TLGS). Including such non-independent data would violate the fundamental assumption of independent samples in meta-analysis and artificially inflate the precision of the pooled estimate.

Therefore, while we ensured all studies from previous reviews were *evaluated*, we did not automatically *include* them all. Each study was rigorously assessed against our pre-defined eligibility criteria, particularly our criterion #4: *"Duplicate publications or studies that used the same dataset."* In such cases, only the publication with the most comprehensive or relevant data was included to ensure the statistical integrity of our synthesis. This process is detailed in our PRISMA flowchart (Figure 1), which notes the exclusion of 12 articles due to "population overlap."

We are confident that our process, combining a comprehensive, multi-database search with a rigorous, criteria-driven evaluation that corrects for the methodological limitations of prior reviews, has resulted in a more robust and reliable evidence synthesis. We have added a brief statement in the Methods (Screening) section to explicitly mention this step: *"The reference lists of previous systematic reviews on this topic were also cross-checked to ensure all relevant primary studies were considered for eligibility."*

**Comment 4.** Recent evaluations have shown possible fundamental errors in interpretation caused by the use of the LFK index as a measure of small-study bias. The use of Egger's test with a higher than conventional threshold of significance (< 0.1 or 0.2 depending on the number of included studies) might be more appropriate. Alternatively selection models may be employed to evaluate for publication bias. The heterogeneity found in prevalence reports from different studies may additionally be a significant factor in identification of small-study bias via plot asymmetry approaches. Smaller studies may indeed be affected by sampling bias (bearing in mind that these studies are usually not population-based) leading to skewed estimates. The suggestion of publication bias when the target effect is prevalence might in turn be less expected as higher prevalence reports might not directly motivate publication. Further evaluation of such a possibility and its incorporation into the manuscript discussion would be appreciated.

- Schwarzer G, Rücker G, Semaca C. LFK index does not reliably detect small-study effects in meta-analysis: A simulation study. Res Synth Methods. 2024 Jul;15(4):603-615. doi: 10.1002/jrsm.1714. Epub 2024 Mar 11. PMID: 38467140.

Answer: Thank you for your valuable explanation and comments. In the present study, in addition to the LFK test, Egger's and Begg's tests were also conducted. The results of all three tests were aligned and indicated statistically significant publication bias. Accordingly, a trim and fill analysis was performed to estimate the number of potentially missing studies. Furthermore, if the esteemed reviewer believes that the results pertaining to the LFK test should be omitted from the article, please kindly advise so that we may proceed accordingly.

**Comment: 5.** Interpretation of meta-regression results when the number if included studies is insufficient (<10 per adjusted variable) might be subject to type II errors. Interpretations arising from subgroup analyses may be more appropriate under such circumstances. For example the proposition that age has no significant effect on the prevalence of MetS seems to be both counterintuitive and not supported by the evidence. Additionally, all subgroups evaluated have also demonstrated a high degree of heterogeneity (inconsistency) in their respective estimates. Therefore other possible sources of heterogeneity should be explored or discussed in review limitations. One such variables to explore are changes in MetS prevalence according to the timeframe of the included studies - as the identified heterogeneity may be in fact indicative of underlying trends in the prevalence of MetS. More recent reports may have similarly benefited from better methodology and reporting leading to the proposition of "higher prevalence in high-quality studies". At minimum a subgroup analysis stratifying studies by study time-frame seems to be warranted.

Answer: We sincerely thank the reviewer for their insightful and constructive feedback. The reviewer's suggestion to consider the potential confounding effect of study timeframe as a source of both heterogeneity and the observed association with study quality is particularly astute. In direct response to this comment, we have performed extensive additional analyses to explore these potential sources of heterogeneity more thoroughly. As suggested, we conducted a subgroup analysis stratifying studies by their publication year, categorizing them into the following timeframes: on or before 2010, 2011-2015, 2016-2020, and after 2020. The results of this analysis have been added to the revised manuscript in the Results section (under "Sources of Heterogeneity") and presented in Table 2.

Furthermore, to comprehensively investigate other potential demographic and healthcare-system-related sources of heterogeneity, we performed additional subgroup analyses based on:

1. Study sample size frame (categorized as very small, small, medium, and large).
2. Healthcare price index of the province where the study was conducted (categorized as high, medium, low), as a proxy for socioeconomic and healthcare access factors.
3. Primary care center density of the province (categorized as high, medium, low), as an indicator of healthcare service availability.

The methodology for these classifications, based on official data from the *Statistics Center of Iran*, has been detailed in the Methods section (under "Statistics").

The results of these new analyses indicated that subgrouping by study year, sample size frame, healthcare price index, and primary care center density did not reveal observable trends or substantially reduce the statistical heterogeneity (I²). Subsequent meta-regression confirmed that the study year, healthcare price index, and primary care center density were not significant sources of heterogeneity. We believe these substantial additions have significantly strengthened the manuscript by providing a more transparent and thorough investigation of heterogeneity, directly addressing the reviewer's valuable concerns.

6. The review title highlight the PRISMA compliance of the manuscript. Appraisal of the quality of evidence (certainty) is an oft-neglected and essential step in ensuring PRISMA compliance. Although the broadly used GRADE approach may not be optimized for meta-analyses of prevalence, several modifications/alternatives may be explored. Use of GRADE with ad hoc modifications is similarly not without merit.

- Yousefifard M, Shafiee A. Should the reporting certainty of evidence for meta-analysis of observational studies using GRADE be revisited? Int J Surg. 2023 Feb 1;109(2):129-130. doi: 10.1097/JS9.0000000000000114.

- Borges Migliavaca C, Stein C, Colpani V, Barker TH, Munn Z, Falavigna M; Prevalence Estimates Reviews – Systematic Review Methodology Group (PERSyst). How are systematic reviews of prevalence conducted? A methodological study. BMC Med Res Methodol. 2020 Apr 26;20(1):96. doi: 10.1186/s12874-020-00975-3.

Kind regards,

Reviewer

Answer: We thank the reviewer for this methodological comment. We extensively considered the application of the GRADE framework for our meta-analysis of prevalence data after your feedback. As the reviewer notes, and as highlighted in the recommended literature by the reviewer, the standard GRADE approach is primarily pertain to interventional studies, with some addressing diagnostic accuracy tests and a limited number concerning prognostic studies, and is not directly applicable to prevalence studies without significant, non-standard modifications. To the best of our search efforts, no official guideline for grading systematic reviews of prevalence studies was identified. In some studies, such research is by default considered of low certainty. Therefore, based on the explanations provided, it has been decided not to include a GRADE assessment. However, to address your concern, and since without a GRADE assessment including the term “PRISMA-Compliant” is not totally correct, we have decided to remove it.

**Reviewer #3**

This manuscript explores an important topic in women’s health by systematically reviewing and quantifying the prevalence of metabolic syndrome (MetS) among Iranian postmenopausal women. The inclusion of recent data from large-scale cohorts and the effort to update and improve on prior reviews are notable strengths. Additionally, the use of subgroup and meta-regression analyses to explore heterogeneity adds analytical depth. However, there are several areas where revisions are necessary to improve clarity, interpretability, and overall scientific rigor.

Abstract

• Improve clarity by explicitly stating the role of study quality, setting, and age as sources of heterogeneity.

Answer: Thank you for your comment. This has been stated on lines 43-44.

• The conclusion could extend beyond screening to mention broader public health or preventive implications.

Answer: Thank you for your suggestion. We have revised the conclusion of the abstract accordingly.

Introduction

• The background is comprehensive but could be more concise and focused on the rationale for this study.

Answer: Thank you for your comment. We have revised this section for to clearly indicate why this study was needed.

• Better articulation is needed regarding how this study improves upon previous reviews. Avoid simply citing inclusion of the PERSIAN cohort—highlight methodological differences more explicitly.

Answer: Thank you for your suggestion. We have explicitly mentioned these methodological differences on lines 104-109.

Methods

• The POLIS criteria should be defined more clearly and not just listed as an acronym.

Answer: Thank you for your comment. We have rewritten and expanded on this acronym on line 124-125).

• While the decision to prioritize the NCEP ATP III definition is acceptable, a brief note on its limitations (e.g., generalizability or sensitivity) would strengthen the justification.

Answer: Thank you for your feedback. In response, we have revised the Methods section (under "Data Extraction") to include a brief discussion of the limitations of our decision. The added text acknowledges that while the NCEP ATP III definition may have specific limitations, such as its fixed waist circumference cut-offs, which might not be perfectly calibrated for all ethnicities, its primary strength in the context of this review is its consistent and predominant use in the existing Iranian literature. This widespread adoption minimizes methodological heterogeneity and facilitates more valid comparisons across the included studies, which was our primary objective.

• Clarify whether study selection and data extraction were done independently by two reviewers.

Answer: Thank you for your comment. These are clarified on lines 161 and 172.

• The scoring thresholds used for the JBI tool (e.g., 7–9 = high quality) should be referenced or justified.

Answer: We thank the reviewer for this comment. The official JBI guidance does not prescribe specific cut-off scores, leaving this judgment to the authors' discretion based on the review's context (Munn, Z., Moola, S., Riitano, D., & Lisy, K. (2014). The development of a critical appraisal tool for use in systematic reviews addressing questions of prevalence. *International journal of health policy and management*, *3*(3), 123–128. <https://doi.org/10.15171/ijhpm.2014.71>).

To provide a clear justification for our chosen thresholds, we have provided established precedent in the field of high-quality prevalence meta-analyses below. The sum of scores from the nine questions determined the overall quality of the paper based on the mentioned scoring system, which has been employed in other high-quality systematic reviews of prevalence data, ensuring our approach is consistent with current best practices in the literature.

1. Soroorikia, S., Kazeminia, M., Qaderi, K. et al. Global prevalence of gastric intestinal metaplasia: a systematic review and meta-analysis. Syst Rev 13, 247 (2024). <https://doi.org/10.1186/s13643-024-02633-x>
2. Bukhari ST, Alhasan HA, Qari MT, Sabbagh HJ, Farsi NM. Prevalence and risk factors of molar incisor hypomineralization in the Middle East: A systematic review and meta-analysis. Journal of Taibah University Medical Sciences. 2023 Aug 1;18(4):696-710.

Results

• The pooled prevalence estimate is clear, but the interpretation should be more cautious given the extreme heterogeneity (I² > 98%).

Answer: Thank you for your comment. We have already addressed this under the “limitations” section on lines 449-453.

• While sensitivity and publication bias analyses were conducted appropriately, their implications deserve more emphasis.

Answer: Thank you for your comment. We have addressed the implications of these analyses in our study on lines 466-471.

• Figure 2 (forest plot) needs to be regenerated in higher resolution. Font size and axis clarity are insufficient for publication.

Answer: Thank you for your feedback. We have replaced our Figures with their bigger and higher resolution exports from the software.

• In Table 2, regional labels (Region 1–5) should be linked clearly to Supplementary File 2 or relabeled more meaningfully.

Answer: Thank you for your feedback. We have linked “Region” in Table 2 to Supplementary File 2 with the “*” sign with explanations below the aforementioned Table.

Discussion

• The discussion covers relevant comparisons across countries and possible mechanisms, but some sections are overly speculative (e.g., attributing prevalence differences to national dietary patterns). These need to be rewritten with more caution and supporting evidence.

Answer: We thank the reviewer for this critical observation. In response, we have revised the relevant section in the Discussion to adopt a more cautious and evidence-based tone. Specifically, we have reframed the narrative from definitive attribution to a discussion of *potential contributing factors*, consistently using hedging language and strengthening the argument with additional citations from comparative nutrition and public health literature. The revised text now more explicitly presents these factors as a plausible, evidence-informed framework for understanding the observed disparities, rather than as conclusive explanations. We believe these changes have significantly tempered the speculative nature of the section, grounding our interpretation more firmly in the existing literature and aligning with the constructive role of a systematic review in identifying potential avenues for future research.

• The biological discussion, while accurate, is too detailed in parts and detracts from the epidemiological focus of the paper.

Answer: We thank the reviewer for their valuable feedback. We agree that an extensive focus on molecular mechanisms could distract from the primary epidemiological findings of the paper. In response, we have significantly shortened the detailed pathophysiological explanation. Furthermore, we have relocated this condensed section to the Introduction, where it more effectively serves to establish the biological plausibility of the association between menopause and metabolic syndrome, thereby providing essential context for the epidemiological investigation that follows. This revision ensures that the Discussion section remains focused on interpreting our results, comparing them with the existing literature, and exploring their public health implications, while still providing the reader with the necessary mechanistic background in a more logical location within the manuscript's narrative.

• The interpretation of meta-regression findings should better acknowledge the limitations of covariate-level analyses with small numbers of studies.

Answer: Thank you for your comment. In direct response to this comment, we have explicitly acknowledge this limitation. We have added a statement that clarifies the exploratory nature of our meta-regression and cautions against overinterpreting both the significant and non-significant associations, framing them as generating hypotheses for future research rather than providing definitive conclusions.

• Greater attention should be given to why study quality and setting were associated with different prevalence estimates.

Answer: Thank you for your comment. In response, we have substantially expanded the Discussion section to provide a more thorough exploration of the potential reasons behind these associations. We now delve into the specific methodological aspects that differentiate high-quality from medium-quality studies, and population-based from institutional settings, linking these directly to potential biases that could affect the prevalence estimates.

Implications and Limitations

• The suggestion to incorporate routine screening into clinical practice is reasonable, but discussion of real-world feasibility, resource constraints, and specific implementation pathways in the Iranian context would add value.

Answer: Thank you for your comment. We have discussed these points on lines 411-428.

• The limitations section is appropriate but could be expanded to discuss misclassification of menopausal status or potential biases in self-reported age at menopause.

Answer: Thank you for your comment. We have expanded limitations section to discuss these issues on lines 438-448.

• The time span across studies may reflect secular trends in MetS prevalence, which is not fully addressed.

Answer: Thank you for your comment. We have now conducted subgroup analysis based on study year.

Language and Presentation

• Numerous grammatical and stylistic issues are present throughout. The manuscript would benefit significantly from professional English editing.

Answer: Thank you for your feedback. We have proofread and rechecked the English language used in our manuscript with a professional language editor and made necessary changes.

• Figures and tables require formatting improvements for consistency and legibility.

Answer: We thank the reviewer for the comment. In the absence of specific points, we have undertaken a comprehensive review of all figures and tables based on standard principles of scientific reporting. The improvements made include:

- Consistency: Ensuring all tables follow an identical format (font, sizing, alignment, and border style).
- Legibility: Increased font sizes in key areas (especially table footnotes and axis labels), adjusted contrast, and simplified layouts to reduce visual clutter.
- Data Clarity: Re-checked all data entries in tables against the original studies to prevent transcription errors. We have also ensured that abbreviations are defined consistently in each table's footnote.
- Standardization: All statistical results are now reported with a consistent number of decimal places throughout the manuscript.

Conclusion

• The conclusion appropriately summarizes the main findings but could be expanded slightly to mention the need for targeted public health policies or future longitudinal research.

Answer: Thank you for your comment. We have expanded this section accordingly.

**Reviewer #4**

1. In the introduction, it would be helpful to also provide a global perspective on the issue. This way, we can see it is particularly important in Iran compared with the global context.

Answer: We thank the reviewer for their insightful comment. Our primary aim was to highlight the critical gap created by the lack of comprehensive, up-to-date, and methodologically sound data within Iran itself, which is essential for informing national and sub-national healthcare policy. Furthermore, a key objective of our analysis was to assess sources of heterogeneity and differences in prevalence within specific Iranian subgroups, a focus that requires a deep dive into the national context first. We have, however, expanded the Introduction section to include a paragraph on lines 76-80, which we believe effectively addresses the spirit of the reviewer's comment by contextualizing our findings on an international scale and compare it to findings in Iran.

2. In the Methods section, it is mentioned that one of the eligible study designs was clinical trial. Could you please explain this.

Answer: We appreciate your comment. Our intention was not to include data from the interventional phases of trials, as those would not reflect the natural population prevalence. Rather, we aimed to include extractable baseline data from the control or intervention groups *before* any trial-specific intervention was administered. At this baseline point, the study population effectively represents a cross-sectional sample that can be assessed for the prevalence of Metabolic Syndrome. This approach is methodologically sound for maximizing data inclusion in prevalence reviews, as it leverages the high-quality baseline assessments often conducted in clinical trials.

3. In the table, there are some abbreviations that do not seem to be commonly used. Please write the full term instead. (for example, instead of 'AAM,' write 'mean menopausal age). Also, please mention the units for all variables and use a consistent number of decimal places throughout the table.

Answer: Thank you for your comment. We have replaced AAM with “Menopausal age”. We have added the units to variables (year to chronological age and menopausal age, and number to sample size). And finally, we have used a consistent number of decimal places throughout the table.

4. Please clarify the institution based and public based definitions in methods section. Also briefly discuss the five regions of Iran in methods section.

Answer: Thank you for your comment. We have elaborated the definitions of institution-based and population-based studies on lines 192-204. Also, we briefly discussed the five regions of Iran on lines 227-229.
