# Supplementary material for: Prevalence of metabolic syndrome among Iranian postmenopausal females: A systematic review and meta-analysis
Source: PLoS One. 2025 Dec 16;20(12):e0338599. doi: 10.1371/journal.pone.0338599 (PMC12707683; doi:10.1371/journal.pone.0338599)
Supplement: Attachment — Submitted filename: Response to Reviewers R2.docx [file pone.0338599.s007.docx]

Dear ***PLoS One*** Editorial Team,

We would like to express our sincere gratitude to you and your team of reviewers for the time and consideration you gave to our manuscript titled “**Prevalence of Metabolic Syndrome among Iranian Postmenopausal Females: a Systematic Review and Meta-Analysis**”. Your expertise and attention to detail were invaluable in helping us to refine our research and present it in a way that is clear and meaningful. We are especially grateful for the helpful comments and feedbacks provided by the reviewer. Reviewer’s insightful critiques and suggestions allowed us to improve the quality of our research and strengthen the overall presentation of our work. We appreciate the opportunity you have given us to share our research with the academic community. Below are the answers to your comments. Furthermore, changes in the manuscript have been highlighted in yellow. We hope you find it helpful.

**Reviewer #3:**

The authors have made clear improvements in response to earlier feedback. The revised manuscript is more coherent, methodologically transparent, and analytically detailed, especially regarding heterogeneity and the influence of study quality and setting. Nevertheless, several important issues remain that should be addressed to strengthen interpretability, contextual depth, and overall presentation quality before publication.

• How might the authors expand the conclusion to better describe how these findings inform public health or preventive strategies in Iran?

Reply: Thank you for your comment. We have revised the conclusion section with specific instructions on how our findings can be translated into public health practice. The reported prevalence rates in our results informs public health policy makers of the MetS burden in this population in order to consider any possible changes in current preventive strategies (which is another title to be studied).

• What are the main limitations of the NCEP ATP III definition in this context—particularly regarding sensitivity and applicability to Middle Eastern populations?

Reply: Thank you for your comment. We have added a paragraph to the Limitation section of the manuscript discussing this matter (line 451-458).

• Given the very high heterogeneity (I² = 98.6%), should the pooled prevalence be interpreted more cautiously as an approximate national estimate rather than a precise figure?

Reply: Thank you for your feedback. The considerable statistical heterogeneity (I² = 98.6%) indicates that the included studies show substantial variation in their individual prevalence estimates, which are influenced by random error. Therefore, the pooled prevalence of 58.42% should indeed not be interpreted as a single, precise national figure but rather as a robust and informative summary estimate of a wide and variable range. This has been mentioned on line 312-316.

• Are there plans to improve the resolution and legibility of the figures, especially the forest and Doi plots, for publication quality?

Reply: Thank you for your feedback. We have exported the images with highest possible quality and resolution from the STATA software.

• Can the biological explanation in the Discussion be further streamlined to maintain focus on epidemiological interpretation and variability across studies?

Reply: Thank you for your suggestion. These biological explanations were relocated to Introduction. The goal was to condense the detailed pathophysiology and directly link it to the "why" behind our study aims. We have further streamlined the biological explanations accordingly.

• What factors might explain the association between study quality, study setting, and prevalence estimates—such as sampling design, representativeness, or analytical rigor?

Reply: Thank you for your comment. We have already discussed this on line 379-399.

• Would it be possible to include a brief discussion on the feasibility of implementing routine MetS screening in Iran, considering resource constraints or access challenges?

Reply: Thank you for your suggestion. We have already discussed this on line 411-430.

• Have the authors considered potential misclassification of menopausal status and bias in self-reported age at menopause, and how these might influence subgroup or meta-regression findings?

Reply: Thank you for your comment. This issue has already been mentioned on line 440-450.

• Could the temporal span of the included studies have introduced secular trends in lifestyle or healthcare access that partly explain heterogeneity?

Reply: Thank you for your suggestion.  Our meta-regression found no significant association between study year and prevalence estimates (P = 0.521). Additionally, a sub-group analysis was performed on study year which indicated no reduction in statistical heterogeneity. These null findings suggest that the effect of temporal trends is likely non-linear and is powerfully confounded by other variables. The strong, independent associations we found for study quality and setting indicate that methodological differences between studies are a more dominant driver of heterogeneity than any underlying temporal trend, effectively masking its signal. Therefore, while secular trends in lifestyle and healthcare are undoubtedly important at a population level, their net effect on our pooled estimate is inconsistent and obscured by the substantial methodological variation across the Iranian research landscape.

• Might it be helpful to mention that future research could use longitudinal or time-stratified analyses to explore temporal changes in MetS prevalence?

Reply: Thank you for your comment. We discussed this on line 482-485.

• Will the authors review tables and figures for consistent formatting and ensure sufficient clarity in labels and legends?

Reply: Thank you for your feedback. We have rechecked tables and figures for typographical errors and made necessary amendments. We have also exported the highest exportable resolution for images from STATA.

• Is another round of professional language editing planned to address the remaining minor grammatical inconsistencies?

Reply: Thank you for your comment. We have proofread the manuscript with an English language editor and made amendments where necessary.

• Finally, would the conclusion benefit from a short statement emphasizing the need for longitudinal monitoring or national-level interventions to address the high burden of MetS among postmenopausal women?

Reply: Thank you for your comment. We have integrated this into the conclusion section.
